# Multiparameter analysis of timelapse imaging reveals kinetics of megakaryocytic erythroid progenitor clonal expansion and differentiation

**DOI:** 10.1038/s41598-022-19013-x

**Published:** 2022-09-28

**Authors:** Vanessa M. Scanlon, Evrett N. Thompson, Betty R. Lawton, Maria Kochugaeva, Kevinminh Ta, Madeline Y. Mayday, Juliana Xavier-Ferrucio, Elaine Kang, Nicole M. Eskow, Yi-Chien Lu, Nayoung Kwon, Anisha Laumas, Matthew Cenci, Kalyani Lawrence, Katie Barden, Shannon T. Larsuel, Fiona E. Reed, Gabriela Peña-Carmona, Ashley Ubbelohde, June P. Lee, Shakthi Boobalan, Yvette Oppong, Rachel Anderson, Colby Maynard, Kaylie Sahirul, Callista Lajeune, Varsha Ivathraya, Tiffany Addy, Patricia Sanchez, Colin Holbrook, Andrew Tri Van Ho, James S. Duncan, Helen M. Blau, Andre Levchenko, Diane S. Krause

**Affiliations:** 1 Department of Laboratory Medicine, Yale School of Medicine, New Haven, CT USA; 2 Yale Stem Cell Center, New Haven, CT USA; 3 Center for Regenerative Medicine and Skeletal Biology, University of Connecticut Health, Farmington, CT USA; 4 Department of Cell Biology, Yale School of Medicine, New Haven, CT USA; 5 Systems Biology Institute, Yale University, New Haven, CT USA; 6 Department of Biomedical Imaging, Yale University, New Haven, CT USA; 7 Department of Pathology, Yale School of Medicine, New Haven, CT USA; 8 Molecular, Cellular, and Developmental Biology, Yale University, New Haven, CT USA; 9 California State University, Long Beach, CA USA; 10 Quinnipiac University, Hamden, CT USA; 11 University of Connecticut, Storrs, CT USA; 12 University of Puerto Rico, Rio Piedras, Puerto Rico; 13 Baxter Laboratory for Stem Cell Biology, Stanford University School of Medicine, Stanford, CA USA

**Keywords:** Cell biology, Imaging

## Abstract

Single-cell assays have enriched our understanding of hematopoiesis and, more generally, stem and progenitor cell biology. However, these single-end-point approaches provide only a static snapshot of the state of a cell. To observe and measure dynamic changes that may instruct cell fate, we developed an approach for examining hematopoietic progenitor fate specification using long-term (> 7-day) single-cell time-lapse imaging for up to 13 generations with in situ fluorescence staining of primary human hematopoietic progenitors followed by algorithm-assisted lineage tracing. We analyzed progenitor cell dynamics, including the division rate, velocity, viability, and probability of lineage commitment at the single-cell level over time. We applied a Markov probabilistic model to predict progenitor division outcome over each generation in culture. We demonstrated the utility of this methodological pipeline by evaluating the effects of the cytokines thrombopoietin and erythropoietin on the dynamics of self-renewal and lineage specification in primary human bipotent megakaryocytic-erythroid progenitors (MEPs). Our data support the hypothesis that thrombopoietin and erythropoietin support the viability and self-renewal of MEPs, but do not affect fate specification. Thus, single-cell tracking of time-lapse imaged colony-forming unit assays provides a robust method for assessing the dynamics of progenitor self-renewal and lineage commitment.

## Introduction

Colony-forming unit (CFU) assays enable researchers to identify the expansion ability and lineage commitment of progenitor cells in vitro. However, traditional CFU assays have several significant limitations. When assessing a colony in a CFU assay, one cannot always be sure that the colony arose from a single cell rather than two different progenitors plated too closely. CFU assays only indicate the outcome, leaving multiple unknowns, including the dynamics of cell behavior (e.g., differential cell death, cell motility, and proliferation rate) prior to and after fate specification. Moreover, traditional CFU assay readouts miss the kinetics of fate decisions that occur during colony formation.

Here, we report an approach for long-term time-lapse imaging of primary human hematopoietic progenitors grown under CFU conditions. The ability to collect data on quantifiable features of progenitors as they form colonies provides a platform for mechanistic studies. For example, this approach can reveal the relationship between cell cycle speed and fate specification suggested by the literature^1^ and can determine the degree to which upstream progenitors undergo symmetric versus asymmetric self-renewal. For the purposes of this study, we define self-renewal by the functional output of the cells such that a bipotent parent cell that divides and yields at least one daughter cell that retains bipotency is considered to have self-renewed. A symmetric self-renewal division in our system is one bipotent MEP giving rise to two bipotent MEP daughter cells. An asymmetric self-renewal division in our system is one bipotent MEP giving rise to one bipotent MEP daughter cell and another daughter cell that is destined to a single lineage. We use this approach to quantitatively assess MEPs as they make their fate decisions in CFU assays. Highly purified MEPs represent a unique state with the potential to differentiate to megakaryocytic (Mk) and erythroid (E) lineages but does not have the potential to differentiate to other myeloid lineages such as monocyte and granulocyte lineages^1–3^. Unipotent Mk progenitors (MkPs) and E progenitors (ErPs) are capable of proliferation and differentiation down the Mk and E lineages, respectively.

To date, researchers have utilized static CFU assays to quantify the bipotency of MEPs and the lineage commitment of MkPs and ErPs^1, 3, 4^. Time-lapse imaging of cells as they form colonies will allow us to address several important unknowns regarding MEPs as they undergo division and fate specification. For example, (1) it is not clear whether MEPs have the ability to undergo self-renewal (either symmetric or asymmetric); (2) our prior studies suggest that cell cycle frequency can toggle MEPs to become Mk- or E-biased but this has not been observed as primary MEP undergo fate specification^1^; and (3) some sorted MEPs form colonies comprised of only Mks or only Es, bringing into question whether these cells are the same as MkPs and ErPs in their differentiation behaviors. In addition, while significant research has investigated the role of transcription factors and other cell-intrinsic factors in MEP lineage commitment^2, 3, 5, 6^, much remains to be learned regarding the contribution of cell-extrinsic factors. For example, researchers have not yet directly assessed the role of thrombopoietin (TPO) and erythropoietin (EPO) in the expansion, survival, and fate specification of highly enriched bipotent MEPs.

In this work, we used automated serial image acquisition of cells over 7 days at time intervals that were optimized to reduce phototoxicity. Lineage tracing by supervised algorithm-mediated cell tracking permits the identification of bipotent cells upstream of lineage-committed progenitors, from which quantifiable features (e.g., division rate, motility, lineage marker expression) of individual clonal progeny can be measured. The data reveal for the first time that MEPs undergo both symmetric and asymmetric self-renewal with variable probabilities over time and cell generation: symmetric self-renewal (expansion) is predominant in the first 3 generations, whereas asymmetric self-renewal is maximal at generation 7 and continues for up to 10 generations. However, all observed MEPs ultimately exhaust, and the frequency of MEP-generating progeny that give rise to only E- or Mk-committed cells is approximately 2:1. Behavioral phenotypic analyses reveal that MkPs are more motile (based on both instantaneous velocity and total distance traveled) than ErPs, with the cell motility of MEPs being intermediate between that of MkPs and ErPs. To further demonstrate the power of this approach in evaluating fate decision, we demonstrate the critical pro-survival, but not fate-instructive, role of TPO and EPO in MEP in vitro. Thus, we have developed a multimodality live-imaging CFU assay approach that addresses cell dynamics and the effects of cell-extrinsic factors on progenitor populations as they undergo expansion and fate specification.

## Results

### Culture optimization

Long-term (7-day) time-lapse microscopy of non-adherent primary hematopoietic progenitors can provide a robust approach for studying single-cell dynamic behavioral phenotypes but poses several technical challenges. The first technical hurdle is the need to stabilize the cells in three-dimensional space to permit accurate cell tracking, and the second hurdle is the need to utilize a cell culture system that can sustain the long-term nutritional requirements of the cells. We resolved both of these technical issues by using a collagen-based semisolid medium (see “Methods”). To restrict the motility of the cells in the z-plane for a consistent focus between cells, we placed a 12-mm^2^ coverslip over the cells in 15 µL of collagen-based medium to create a sandwich with a depth of roughly 30 µm. To reduce the rate of evaporation, we added additional semisolid medium to cover the coverslip and well bottom.

### In situ staining of colonies in semisolid media

**Figure 1:**
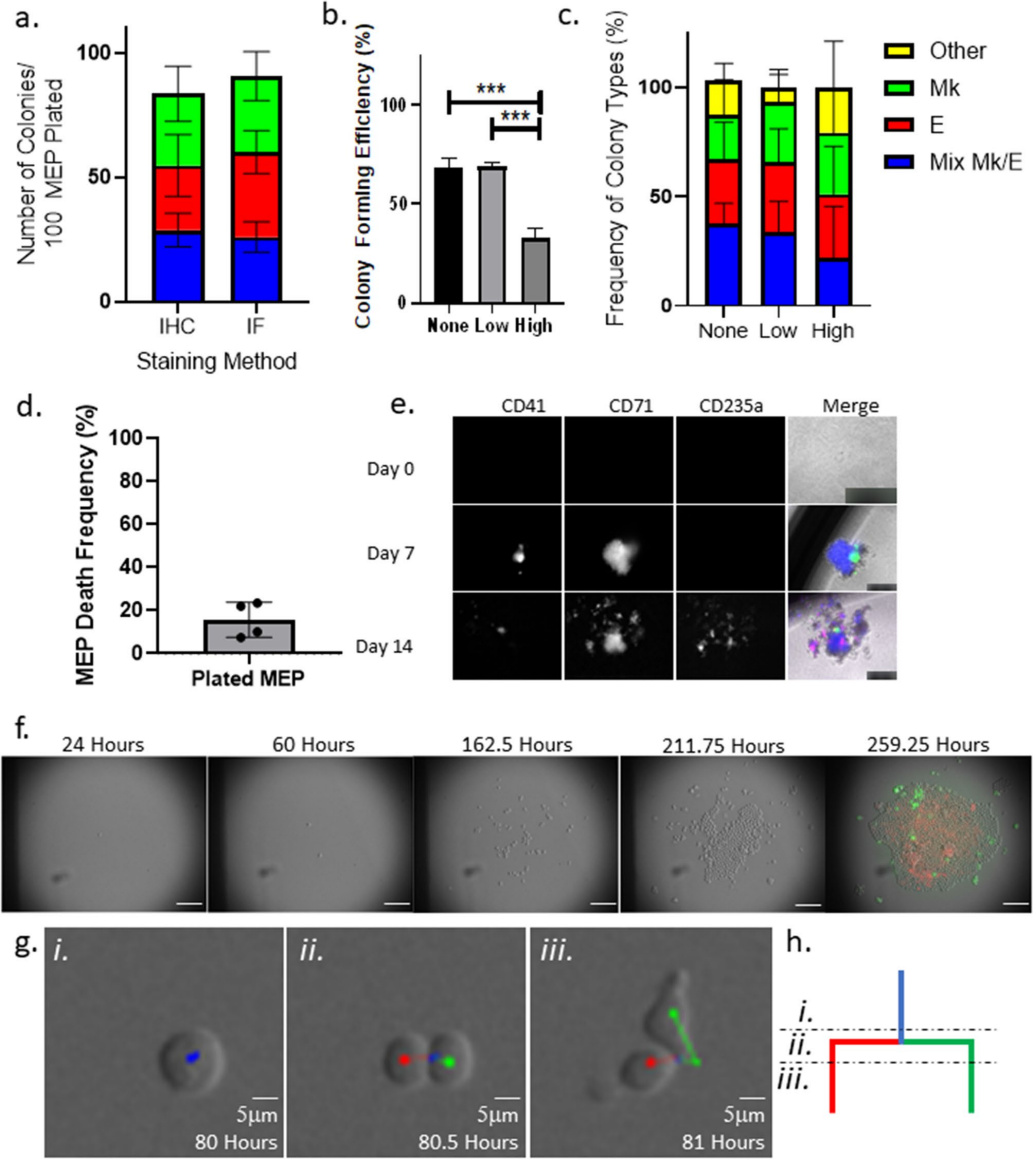
Approach setup. (**a**) In situ immunofluorescence staining detects a similar number and ratio of colony types derived from individual MEPs. MEP CFU assays grown for 14 days were stained using either the traditional immunohistochemistry protocol or the in situ immunofluorescence protocol. Colonies were categorized based on the presence of cells stained with antibodies against lineage-specific markers. Three colony types were scored: Mk/E mixed colonies (blue), E-only colonies (red), Mk-only colonies (green). The average absolute colony counts per 100 plated MEPs by type are presented in stacked bar graphs. n ≥ 5. Mean and SD indicated. (**b**) MEP phototoxicity is avoided by reducing initial imaging frequency. Colony forming efficiency of MEPs grown for 7 days, calculated by the number of colonies observed out of the total plated cells is represented in the bar chart according to image acquisition settings. “None” represents colonies grown in the absence of imaging. “Low” represents colonies grown in the absence of imaging for the first 24 h, followed by imaging every 2 h for the next 36 h, and finally imaging every 10 min for the duration of colony growth. “High” represents colonies grown in the absence of imaging for the first 24 h followed by imaging every 10 min for the duration of colony growth. n ≥ 3; ***p < 0.01. Mean and SD indicated. (**c**) Low imaging frequency does not alter ratio of colony types grown from individual MEPs. Colony count normalized to the total number of colonies grown in each of the acquisition conditions. n ≥ 3; ***p < 0.01. Mean and SD indicated. (**d**) Frequency of MEP death with low imaging frequency. MEP death frequency was calculated as the number of MEPs that failed to give rise to a colony as observed in timelapse acquisitions normalized to the total number of imaged MEPs. n = 4. Mean and SD indicated. (**e**) Representative clonally growing MEPs stained with fluorescently conjugated anti-CD41, anti-CD71, and/or anti-CD235a during colony growth. Individual MEPs and colonies grown from individual MEPs were stained in situ with fluorescently conjugated antibodies against lineage markers at day 0, 7, and 14 of the CFU assays. Representative images of cells and colonies at the respective time points are shown for each channel (marker) and merged. Scale bars are set to 50 µm for day 0 and 250 µm for days 7 and 14. (**f**) Representative time-lapse sequence of an individual MEP forming a colony and in situ stained with anti-CD41 and anti-CD71. Selected time points during the acquisition are shown to demonstrate colony growth from an individual MEP. Scale bar = 100 μm. (**g**) Tracking MEP mitotic events with the Baxter algorithm. A representative image sequence capturing a tracked bipotent progenitor (i) undergoing cytokinesis (ii) and separating into daughter cells (iii). Scale bar = 5 μm. (**h**) Example of lineage tree branching reflecting a mitotic event. Illustration of lineage branching built by single-cell tracking of time-lapse sequences. Blue line represents a bipotent progenitor. Red line represents an E-destined daughter cell. Green line represents an Mk-destined daughter cell. Subsections of the lineage branch correspond with the representative tracked cells in (**g**).

Another technical hurdle in tracing the lineage of primary human hematopoietic progenitors is the lack of an endogenous fluorescent lineage reporter. Previous approaches for identifying colony types based on the presence of lineage-restricted progeny have relied on morphological analyses of brightfield microscopic images, which require a highly trained eye and can result in discrepancies between independent observers. We previously reported an immunohistochemistry-based approach for staining colonies with antibodies derived from disparate species that bind lineage-specific markers (CD235a for E and CD41 for Mk) to reveal the lineages present within colonies after physical transfer of semisolid media to a slide, evaporation, and fixation. While this colorimetric approach allows simultaneous visualization of two lineages, it is time-consuming, is limited in the number of lineages than can be detected, and causes mechanical disturbances to the cultures, which risks disrupting colonies and thereby altering the observed colony numbers. To resolve these limitations, we implemented an in situ staining approach: we stained colonies in situ by directly adding fluorescently conjugated antibodies against CD235a and CD41 to the cultures during time-lapse microscopy. We confirmed that this direct immunofluorescent approach results in colony counts and outcomes similar to those of the previously reported immunohistochemistry method (Fig. 1a).

### Light exposure optimization

To enable accurate cell tracking, we attempted to image the cells in brightfield with an exposure time of 75 ms every 10 min for 7 days; however, significant cell death occurred within the first 2 days of imaging, resulting in failure to form colonies in 50% of cells imaged compared with unimaged controls (Fig. 1b). Reducing the exposure time decreased the image quality such that it became difficult to discern the boundaries of individual cells. We found that the first MEP division occurs between 24 and 48 h of culture (Supplemental Fig. 1a), therefore, to reduce phototoxicity to the primary cells, we initially imaged the cells only once during the first 24 h, followed by imaging every 2 h with a 75 ms exposure time over the next 36 h (60 h in culture). For time points beyond 60 h in culture, the rapid motility and high division frequency of the cells necessitated 10 min acquisition intervals in order to reach the required confidence threshold in the cell-tracking parameters described below. We found that after the first 60 h of less-frequent imaging, the cells could tolerate 75 ms exposure to light every 10 min for the remainder of the acquisition period. This approach restored the colony-forming efficiency to match that of unimaged control cultures (Fig. 1b) while still facilitating single-cell tracking. Importantly, the image acquisition did not cause a change in colony-type distribution (Fig. 1c). Among all of the MEPs imaged, 15.7 ± 4.1% died before giving rise to progeny (Fig. 1d), consistent with the colony-forming efficiency observed in static CFU assays. Thus, having overcome the technical hurdles to long-term time-lapse imaging of primary hematopoietic cells, we proceeded with this acquisition frequency and exposure time protocol to observe colony formation of MEPs.

### Choice of lineage markers to identify Mk- and E-committed progeny

Due to the rapid expansion of E-destined progeny in growing colonies, accurate tracking of individual cells was only possible for up to 7 days of colony growth. At this time point, however, CD235a was not yet detectable for many E-destined cells that ultimately became E-committed. Therefore, we investigated alternative antibodies to discriminate E-destined progeny from Mk-destined progeny downstream of MEPs. We tested antibodies against CD36, CD44, and CD71 on cells from plated MEPs at multiple time points, with the “ground truth” being determined at day 14 with anti-CD235a (Supplemental Table 1). Neither CD36 nor CD44 uniquely marked E-committed progeny at day 7 (Supplemental Fig. 1b and data not shown). Bright CD71 staining proved to be a unique marker for early E-committed progeny; we observed bright staining by day 7 post-plating only in cells that were later marked with CD235a by day 14 (Fig. 1e).

### Cell segmentation and tracking to build lineage trees

To construct a lineage tree of all progeny from each time-lapse-imaged single CFU, one must be able to track each individual cell as it forms from mitosis, follow its trajectory, and mark its division. The first step is to stack the images based on time to create a continuous movie (Fig. 1f). Tracking the individual cells requires two important aspects: (1) accurate cell segmentation, where individual cell boundaries can be recognized, and (2) accurate cell tracking, where appropriately segmented cells are linked from frame to frame over time. While cell tracking is intuitive for humans, automation of these steps requires model optimization to minimize human input.

**Figure 2:**
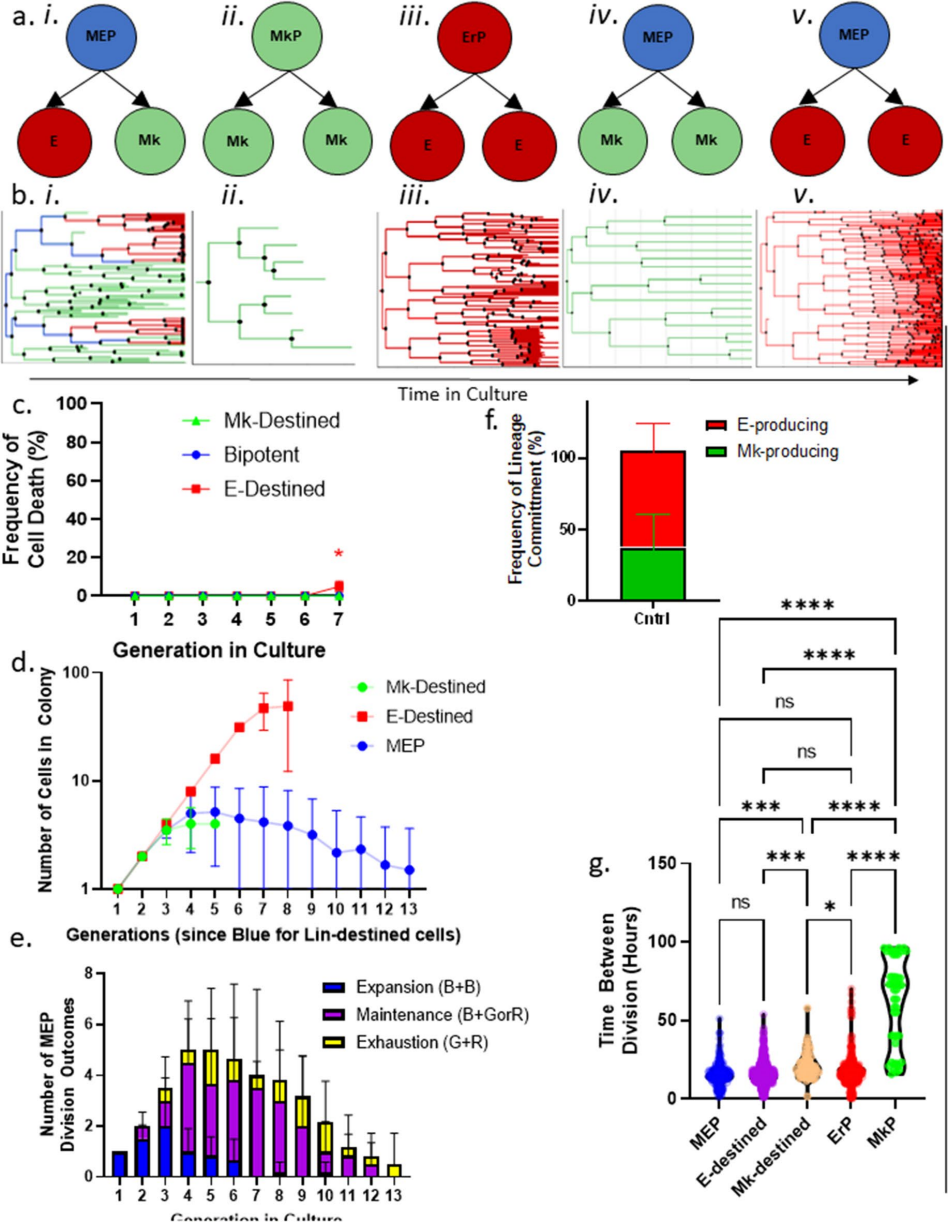
Growth and differentiation. (**a**) Schematic representations of the sorted progenitors and resulting colony types. (i) Sorted MEPs that give rise to an Mk/E mixed colony. (ii) Sorted MkPs that give rise to an Mk-only colony. (iii) Sorted ErPs that give rise to an E-only colony. (iv) Sorted MEPs that give rise to an Mk-only colony. (v) Sorted MEPs that give rise to an E-only colony. (**b**) Corresponding representative lineage trees for each colony output. Blue lines represent MEPs, red lines represent E-destined cells, and green lines represent Mk-destined cells. Italic roman numerals correspond with (**a**). (**c**) Cell death rate by cell state in control culture conditions. Blue points represent bipotent cells (n = 222). Red points represent E-destined cells (n = 2035; p < 0.017). Green points represent Mk-destined cells (n = 165). Mean and SD indicated. (**d**) Expansion rates of cell types in time-lapse CFU assays. Cell number at each generation by cell state: MEP (blue), Mk-destined (green), and E-destined (red) cells grown in control conditions over 13 divisions (n = 6 movies). Mean and SD indicated. (**e**) Division outcomes by generation in culture. Absolute number of MEP division outcomes by generation in culture categorized as expansion (blue: 1 MEP → 2 MEP), maintenance (purple: 1 MEP → 1 MEP + 1 E- or Mk-destined progenitor), or exhaustion (yellow: 1 MEP → 1 E-destined progenitor + 1 Mk-destined progenitor) (n = 6 movies). Mean and SD indicated. (**f**) Frequency of MEP lineage commitment. Frequency of lineage commitment was calculated as the number of E- or Mk-destined daughter cells produced by MEPs in control timelapse CFU conditions (n = 6 movies). Mean and SD indicated. (**g**) Lifespan of cell states. Lifespan of cells categorized by state as defined in the timelapse CFU assays was measured by time between divisions in hours of bipotent MEP (blue; n = 188), E-destined (purple; n = 964), Mk-destined (orange; n = 49), committed ErP (red; n = 505), and committed MkP (green; n = 47) cells. *p < 0.05, **p < 0.01, ***p < 0.001, ****p < 0.0001, *ns* not significant.

The Baxter algorithm (version 1.5.3) allows for customized cell segmentation and tracking settings. We set the segmentation settings (Supplemental Fig. 2) to account for tightly packed cells in a colony by limiting the expected size of each cell and variance in pixel intensity in the brightfield images and incorporate a segmentation watershed to define cell boundaries. The Baxter algorithm also uses probabilistic inference based on specific tracking settings that limit the maximum distance that a cell can travel between frames to predict which cell matches a given cell from the previous time frame with high accuracy (Supplemental Fig. 3)^7–11^. Using the Baxter algorithm, we color-coded the cells according to their state as follows: (1) blue cells are upstream of both Mk and E daughters (bipotent), (2) green cells (Mk-destined) are upstream of Mk-only progeny, and (3) red cells (E-destined) are upstream of E-only progeny (Fig. 1f–h; Supplemental Video 1). Using this approach, we built lineage trees for individual MEPs producing mixed Mk/E, Mk-only, and E-only colonies (Fig. 2a,b i, iv, v). For comparison with the inferred cell state (Mk-destined and E-destined), we also built lineage trees from “ground truth” prospectively sorted Mk-committed (MkP) and E-committed (ErP) cells, which were functionally validated by traditional CFU assays^3^ to grow unilineage colonies (Fig. 2a,b ii, iii). Based on quantitative metrics as presented in detail below, MEP that give rise to Mk-only and E-only colonies are quite similar to MEP that give rise to Mk/E mixed colonies and are distinct from already committed MkP and ErP.

**Figure 3:**
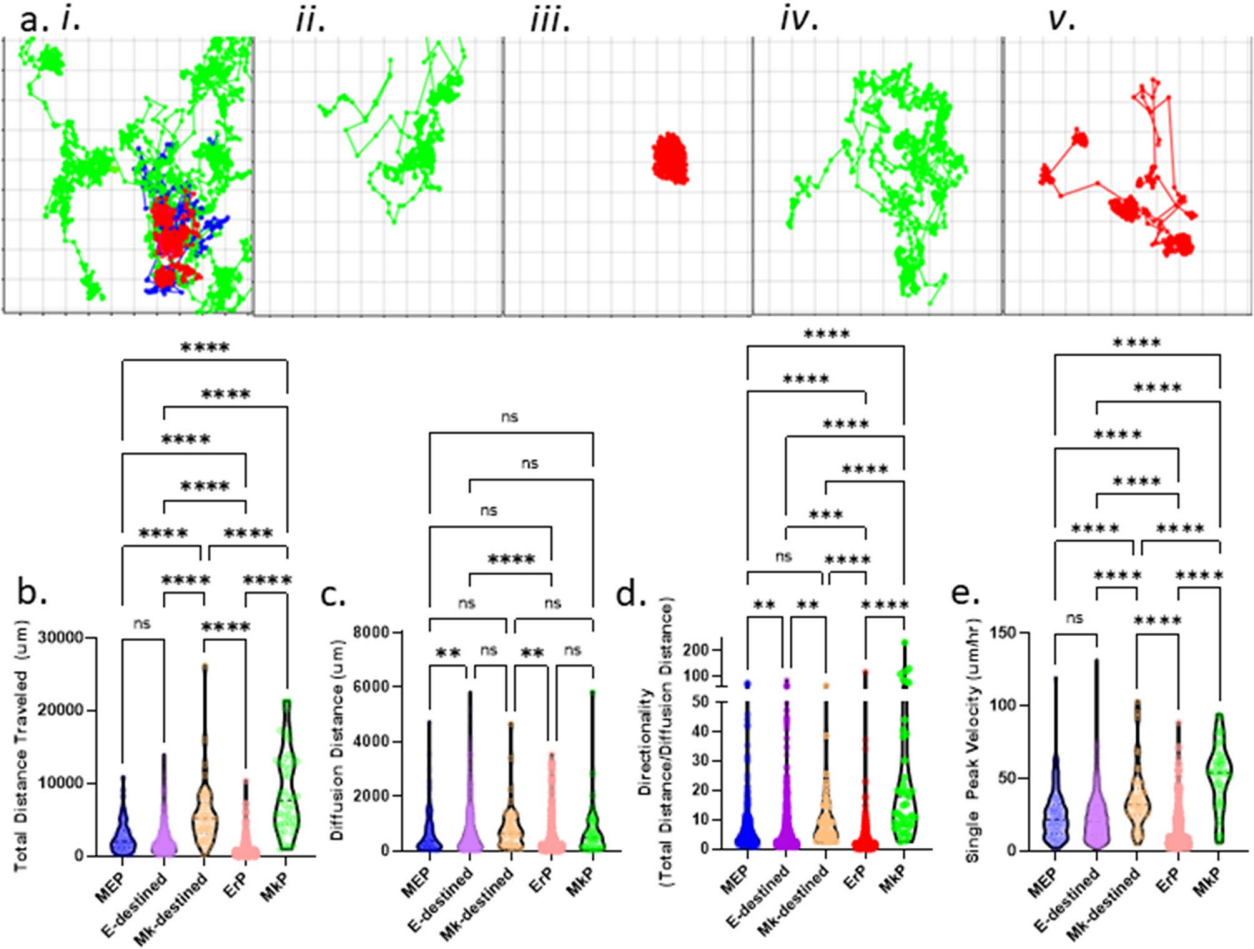
Motility is a differential behavioral phenotype between progenitor cells undergoing state transitions. (**a**) Corresponding representative trajectory maps for each colony output. Cell trajectories were generated based on the x,y coordinates of cells in time-lapse CFU assays. Blue trajectories represent MEPs, red trajectories represent E-destined cells, and green trajectories represent Mk-destined cells. Italic roman numerals correspond with Fig. 2a. (**b**) Total distance traveled by cell state. Total distance was measured as the sum of the distance traveled by an individual cell between time points for the entire lifetime of the bipotent MEP (blue; n = 188), E-destined (purple; n = 964), Mk-destined (orange; n = 49), committed ErP (red; n = 505), and committed MkP (green; n = 47) cell. *p < 0.05, **p < 0.01, ***p < 0.001, ****p < 0.0001, *ns* not significant. (**c**) Diffusion distance traveled by cell state. Diffusion distance was measured as the shortest distance between the starting and ending position of the bipotent MEP (blue; n = 188), E-destined (purple; n = 964), Mk-destined (orange; n = 49), committed ErP (red; n = 505), and committed MkP (green; n = 47) cell at the end of their lifespan. *p < 0.05, **p < 0.01, ***p < 0.001, ****p < 0.0001, *ns* not significant. (**d**) Directionality of motility by cell state. Directionality was calculated by the ratio of total distance over diffusion distance of bipotent MEP (blue; n = 188), E-destined (purple; n = 964), Mk-destined (orange; n = 49), committed ErP (red; n = 505), and committed MkP (green; n = 47). *p < 0.05, **p < 0.01, ***p < 0.001, ****p < 0.0001, *ns* not significant. (**e**) Peak velocity by cell state. Peak velocity is reported as the single highest distance traveled between two time frames over the time between frames for the bipotent MEP (blue; n = 188), E-destined (purple; n = 964), Mk-destined (orange; n = 49), committed ErP (red; n = 505), and committed MkP (green; n = 47) cell. *p < 0.05, **p < 0.01, ***p < 0.001, ****p < 0.0001, *ns* not significant.

### Characterization of individual cells within each colony

We tracked each downstream cell within all of the colonies until it divided, died, visibly began endomitosing, or was no longer trackable due to crowding. We ended acquisitions once we confirmed the lineage of the cell based on cell surface marker expression. Cell tracking allows for quantitation of division types (symmetric self-renewal, maintenance self-renewal, or differentiation), division rates, and motility over time at the single-cell level.

This type of dynamic insight into individual progenitor cells as they make fate decisions significantly adds to the traditional static CFU assay. By providing information on the number and rate of cell divisions and motility in each state (bipotent MEPs, Mk- or E-destined MEPs, and unipotent MkPs and ErPs), we can analyze these progenitor cells throughout differentiation. Note that by eye, when cells were tracked in individual colonies, differences in the number and rate of cell divisions between the cell states were apparent. Thus, we performed quantitative analyses of cell movement and division rates in the context of fate specification as described below.

### Analysis of cell growth and differentiation of time-lapse imaged MEPs

To identify unique cell states between closely related progenitors, we quantified the cell death, division rate, motility, and division outcome of every cell downstream of an MEP. These metrics allowed us to calculate the cell death rate (Fig. 2c), expansion capacity (Fig. 2d), division outcomes by generation (Fig. 2e,f), and lifetime of each cell (Fig. 2g).

With the optimized acquisition settings described above, we observed very low cell death rates in the time-lapse movies, with a slightly higher death rate (< 5%) in E-destined daughter cells compared with Mk-destined daughter cells starting in the sixth generation downstream of the MEP (Fig. 2c).

### Analysis of expansion potential by cell state

The proliferative potential showed striking differences between Mk- and E-destined progenitors. E-destined daughter cells had the greatest expansion, with exponential growth for at least seven generations, after which we could no longer discern individual cells to continue tracking (Fig. 2d). Bipotent MEPs had a shorter period of exponential expansion, with symmetric self-renewal (one blue cell giving rise to two blue cells) peaking between the fourth and fifth generation in culture. The MEPs were then capable of self-renewing asymmetrically to maintain bipotency for more than 13 generations (Fig. 2d). Mk-destined progeny had the lowest expansion potential, undergoing symmetric self-renewal (one green cell giving rise to two green cells) for an average of 2.5 generations up to a maximum of 5 generations before terminally maturing into endomitosing megakaryocytes (Fig. 2d).

Our data indicate for the first time that primary human MEPs are capable of self-renewal, undergoing a transient expansion phase that culminates in exhaustion as late as 13 generations in culture (Fig. 2e; Supplemental Fig. 4). The lineage output of MEPs is significantly skewed toward the E lineage, partly because of the higher expansion capacity of E-destined progeny, but also because; MEPs generate twice as many E-destined daughter cells as Mk-destined daughter cells (Fig. 2f).

**Figure 4:**
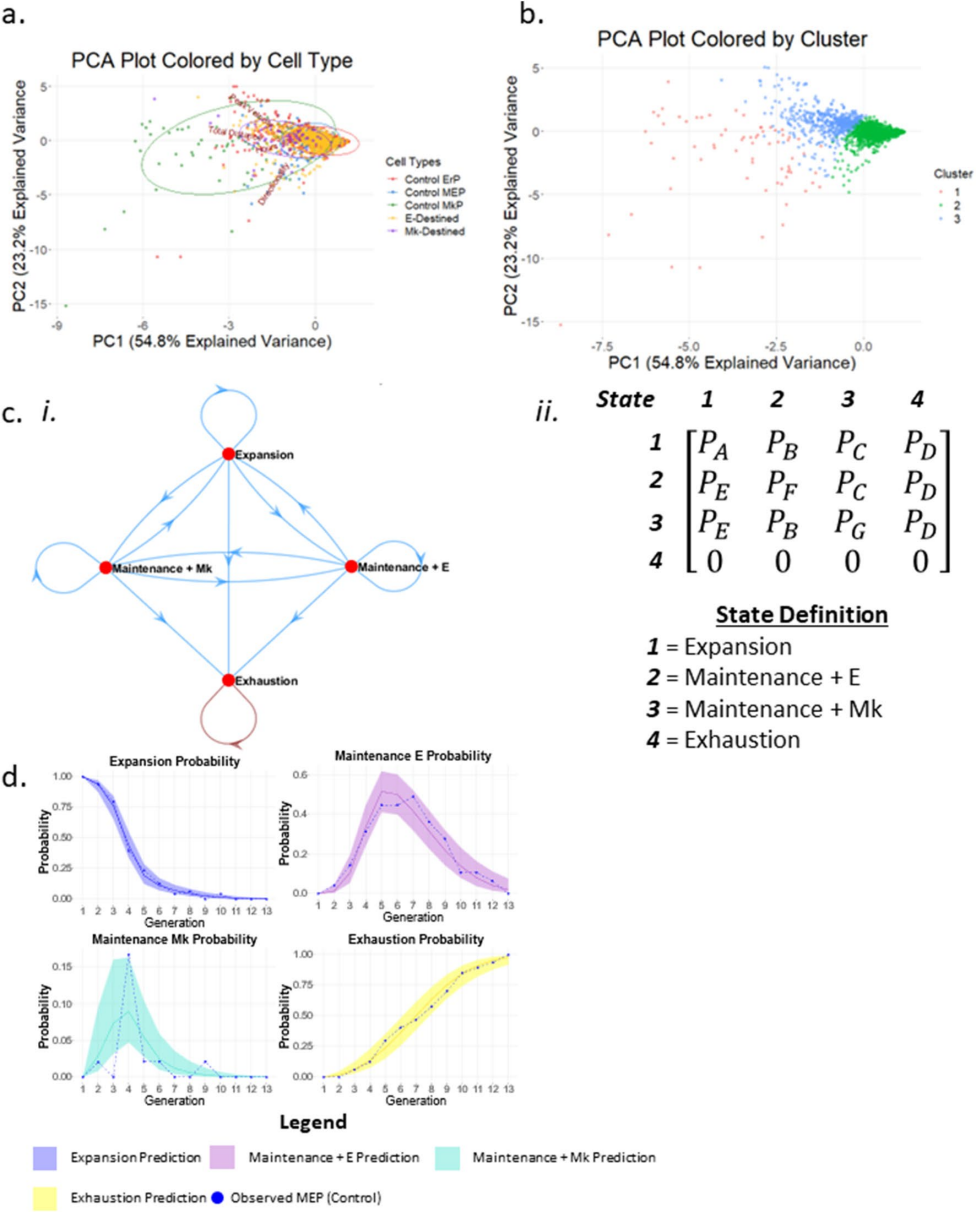
Quantitative behavioral phenotypes predict cell state. (**a**) Clustering of cells based on multi-dimensional analysis. Individual cells are graphed on PCA plots based on all quantified behavioral phenotypes (peak velocity, total distance, lifespan, and directionality) and color coded based on cell state: bipotent MEP (blue), E-destined (orange), Mk-destined (purple), committed ErP (red), and committed MkP (green). (**b**) k-means clustering of PCA plots. Three clusters were generated by k-means clustering. (**c**) Markov chains depicting cell state transitions of MEPs in CFU culture conditions. (i) Markov chains most accurately encompass the cell state transitions MEPs were observed to undergo. States include expansion, maintenance + E, maintenance + Mk, and exhaustion. (ii) The matrix used to derive the mathematical model to predict MEP state transitions. (**d**) Probability of MEP state transitions. The probability that MEPs will undergo four division types (expansion, maintenance + E, maintenance + Mk, exhaustion) by generation in culture is represented by the solid lines in each graph with a 95% confidence interval represented by the colored shadowed regions. Actual observed frequencies of each division outcome by generation are depicted by the dotted lines.

**Table 1:**
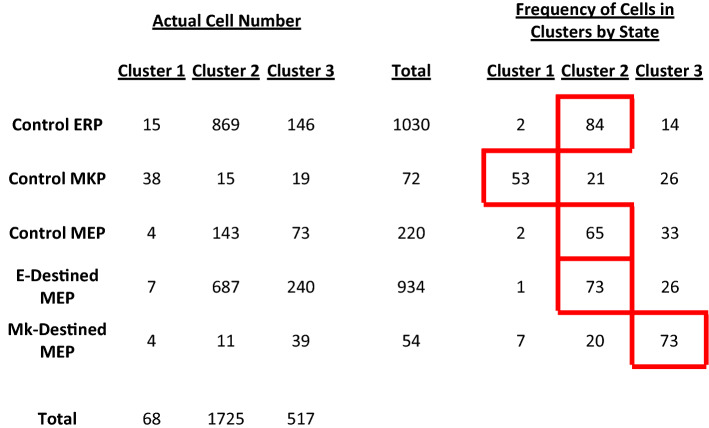
Frequency of cell states located within designated cluster. Progenitors were categorized by cell state and the number of cells per state were counted according to the PCA clusters. Actual cell numbers are on the left, and percentages are on the right. The predominant cluster for each cell state is outlined in red.

MEPs, ErPs, and MkPs all start in culture with similarly slow divisions, which is likely an artifact of isolating the primary cells and seeding of primary cells into in vitro cultures, but the division rate increases disparately in a manner that correlates tightly with different cell states. On average, MEPs divide every 15.8 h, whereas MkPs have an average cycle length of 35.7 h, ErPs have an average cycle length of 10.2 h, E-destined MEP daughters have an average cycle length of 15.5 h, and Mk-destined MEP daughters have an average cycle length of 25.5 h, indicating that the cell cycle rate increases as cells commit to the E lineage and decreases as cells commit to the Mk lineage (Fig. 2g).

### Quantitation of motility

The use of time-lapse imaging and cell tracking to build a lineage history uniquely enables us to add motility measurements to multimodal single-cell analyses. We exported cell trajectories for all cells within a forming colony from the Baxter algorithm (Fig. 3a i–v), and for each cell in the colony, we determined the total distance traveled (Fig. 3b), diffusion distance (absolute distance between starting and ending position; Fig. 3c), directionality (calculated as the ratio of total distance over diffusion distance; Fig. 3d; Supplemental Fig. 5a), and peak velocity (Fig. 3e; Supplemental Fig. 5b). The cell trajectories tightly correlate with cell state. The ground-truth ErPs have the lowest motility; trajectory mapping over their lifetime reveals that these cells remain relatively stationary, with tight colonies of closely related cells (Fig. 3a iii). In contrast, the trajectories of ground-truth MkPs demonstrate much higher motility early in culture, with a deceleration as the cells enter endomitosis (Fig. 3a ii). The trajectories of MEPs are similar to those of early MkP (Fig. 3a i). This trajectory analysis enables us to compare MEPs giving rise to E-only colonies versus ErPs, and MEPs giving rise to Mk-only colonies versus MkPs. Here, we find that the motility data separate progenitors based on cell state because E-destined progeny of bipotent cells have a much higher total distance traveled and peak velocity than lineage-committed ErPs (Fig. 3a; Supplemental Fig. 5b). Mk-destined progenitors exhibit an increased velocity during differentiation and a decreased velocity during terminal maturation whereas E-destined progenitors show a decreased velocity during commitment and maturation (Fig. 3e). These striking cell-state-dependent differences in motility patterns, where MkPs exhibit the most and ErPs exhibit the least motility, suggest inherent differences between MEPs that give rise to E-only or Mk-only colonies and sorted ErP or MkP cells. These data further support the hypothesis that sorted MEPs that give rise to E-only colonies are not in fact contaminating ErPs, but rather a bipotent cell that has a probability of forming progeny of only the E lineage. This behavioral phenotypic difference between lineage-destined and lineage-committed progenitors also corroborates single-cell transcriptomic analyses demonstrating discrete cell states in immunophenotyped cells^1^.

**Figure 5:**
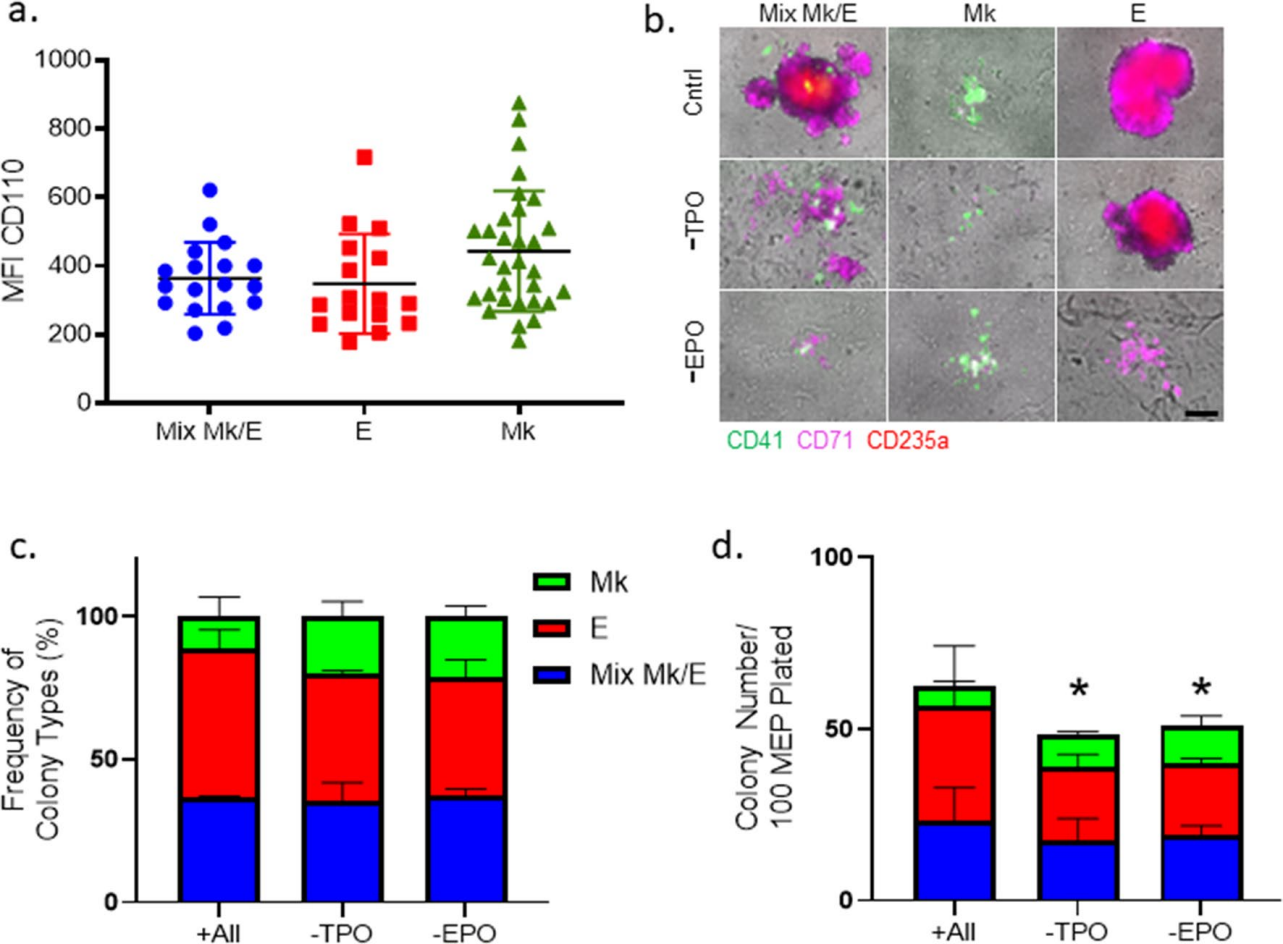
Thrombopoietin and erythropoietin do not instruct MEP lineage commitment. (**a**) Index sorting and analysis of TPO receptor, c-Mpl (CD110). MEPs were index sorted into 96-well plates and grown in CFU assay conditions for 14 days. Colony type outcome was correlated with expression of CD110 at the time of sorting/plating. n = 66. Mean and SD indicated. (**b**) Representative colonies grown from individual MEPs in control, -TPO, and -EPO conditions. Mk/E mix, E-only, and Mk-only colonies were observed in control, -TPO, and -EPO CFU conditions at 14 days. Cells within the colony were stained in situ with CD41 (green), CD71 (magenta), and CD235a (red). Scale bar = 250 μm. (**c**) Frequency of colony types observed in control, -TPO, and -EPO CFU assays. Colony counts (Mk/E mix in blue, E-only in red, and Mk-only in green) were normalized to total colonies grown in control, -TPO, and -EPO conditions. n = 4. Mean and SD indicated. (**d**) Colony counts of MEPs grown in control, -TPO, and -EPO CFU assays. Colony counts (Mk/E mix in blue, E-only in red, and Mk-only in green) are reported out of 100 plated MEPs grown in control, -TPO, or -EPO CFU conditions. n = 4, *p < 0.05. Mean and SD indicated.

### Progenitor clustering by behavioral phenotype

To determine if proliferative rate and motility alone can predict cell state, we conducted PCA analysis to reduce multiple dimensions (lifespan, velocity, distance, and directionality) and visualize potential cell state clusters (Fig. 4a). We performed clustering through a combination of unsupervised and supervised approaches. We first determined the optimal number of clusters through hierarchical clustering, in which the cells were partitioned by a function to identify *k* = 3 clusters (Supplemental Fig. 6). We then applied this value of *k* to conduct *k*-means clustering to visualize cell-state clusters (Fig. 4b). Although we did not observe discrete clustering among bipotent MEPs, Mk-destined cells, E-destined cells, and control MkPs and ErPs, we observed a 53% enrichment of MkPs in cluster 1, whereas cluster 2 exhibited an 84% enrichment of ErPs. Cluster 2 also contains 65% of the bipotent cells, indicating that ErPs more closely mimic MEPs in cycling and motility than MkPs. Interestingly, as Mk-destined cells transition from bipotency to Mk commitment, we observe a 72% enrichment in cluster 3, demonstrating that the proliferative rate and motility of these cells gradually shift toward that of committed MkPs. Similarly, E-destined cells gradually shift toward committed ErPs, with 73% enrichment in cluster 2 (Table 1).

**Figure 6:**
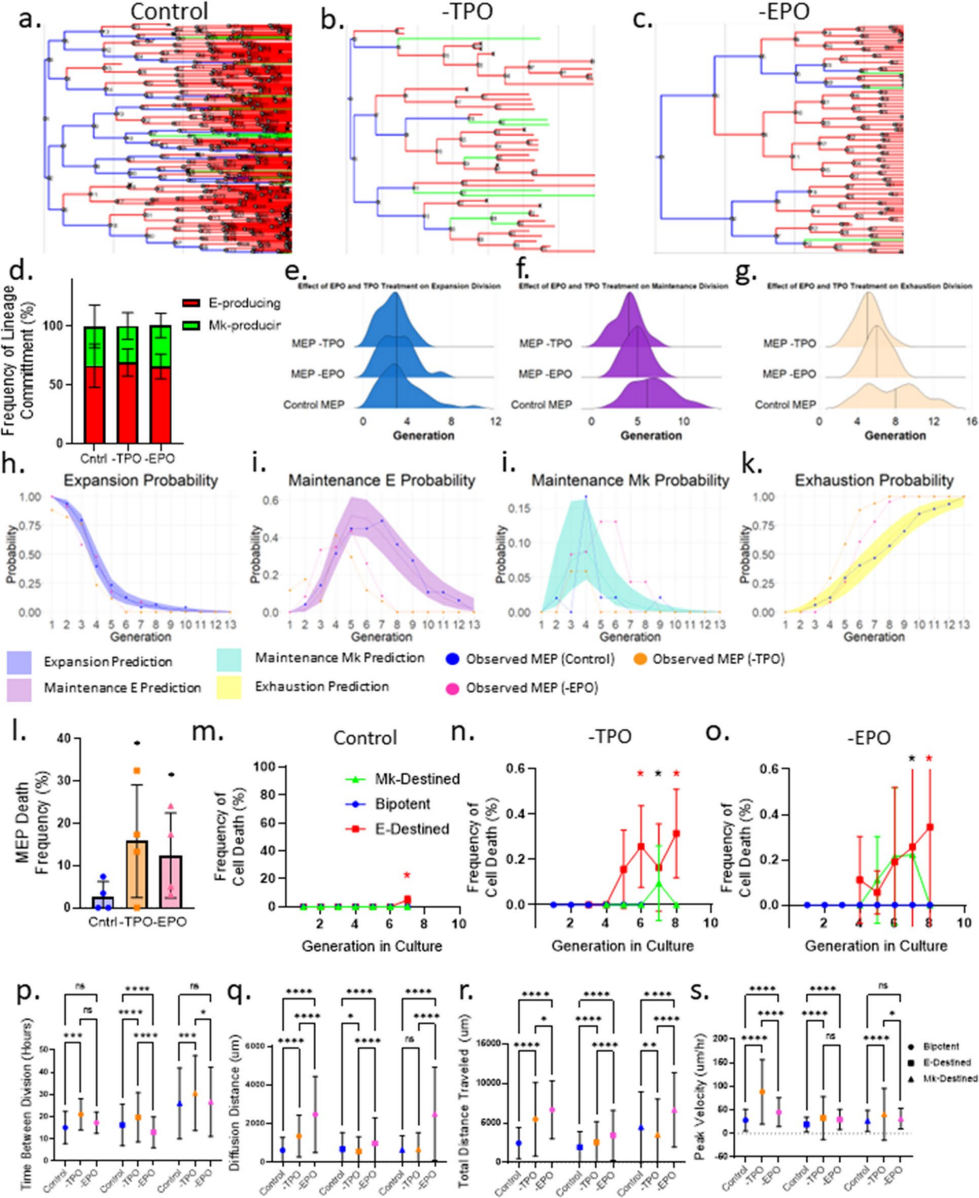
Thrombopoietin supports MEP self-renewal as well as survival of MEPs and all downstream lineage progenitors. (**a**) Representative lineage tree of Mk/E mixed colony grown in control conditions. Blue lines represent MEPs, red lines represent E-destined cells, and green lines represent Mk-destined cells. (**b**) Representative lineage tree of Mk/E mixed colony grown in -TPO conditions. Blue lines represent MEPs, red lines represent E-destined cells, and green lines represent Mk-destined cells. (**c**) Representative lineage tree of Mk/E mixed colony grown in -EPO conditions. Blue lines represent MEPs, red lines represent E-destined cells, and green lines represent Mk-destined cells. (**d**) Frequency of MEP lineage commitment. Frequency of lineage commitment was calculated as the number of E- or Mk-destined daughter cells produced by MEPs normalized to 100% in control, -TPO, and -EPO timelapse CFU conditions (n ≥ 3 movies). Mean and SD indicated. (**e**) Distribution of MEP expansion divisions over time in culture. Distribution of MEP expansion divisions in control, -TPO, or -EPO conditions. The mean is represented by a solid vertical line (n ≥ 3 movies). (**f**) Distribution of MEP maintenance divisions over time in culture. Distribution of MEP expansion divisions in control, -TPO, or -EPO conditions. The mean is represented by a solid vertical line (n ≥ 3 movies). (**g**) Distribution of MEP exhaustion divisions over time in culture. Distribution of MEP expansion divisions in control, -TPO, or -EPO conditions. The mean is represented by a solid vertical line (n ≥ 3 movies). (**h**) Probability of MEP expansion divisions. The probability that MEPs will undergo expansion divisions by generation in culture is represented by the solid line with a 95% confidence interval represented by the colored shadowed region. Actual observed frequency of expansion division outcome by generation is depicted by the dotted line. (**i**) Probability of MEP maintenance + E divisions. The probability that MEPs will undergo maintenance + E divisions by generation in culture is represented by the solid line with a 95% confidence interval represented by the colored shadowed region. Actual observed frequency of maintenance + E division outcome by generation is depicted by the dotted line. (**j**) Probability of MEP maintenance + Mk divisions. The probability that MEPs will undergo maintenance + Mk divisions by generation in culture is represented by the solid line with a 95% confidence interval represented by the colored shadowed region. Actual observed frequency of maintenance + Mk division outcome by generation is depicted by the dotted line. (**k**) Probability of MEP exhaustion divisions. The probability that MEPs will undergo exhaustion divisions by generation in culture is represented by the solid line with a 95% confidence interval represented by the colored shadowed region. Actual observed frequency of exhaustion division outcome by generation is depicted by the dotted line. (**l**) Frequency of MEP death in control, -TPO, and -EPO CFU conditions. MEP death frequency was calculated as the number of MEPs that failed to give rise to a colony as observed in time-lapse acquisitions normalized to the total number of imaged MEPs for control, -TPO, and -EPO conditions (n ≥ 3, *p < 0.05). Mean and SD indicated. (**m**) Cell death rate by cell state in control culture conditions. Blue points represent bipotent cells. Red points represent E-destined cells (*p < 0.05). Green points represent Mk-destined cells (n = 6 movies). Mean and SD indicated. (**n**) Cell death rate by cell state in -TPO culture conditions. Blue points represent bipotent cells. Red points represent E-destined cells (*p < 0.05). Green points represent Mk-destined cells (*p < 0.05, n = 4 movies). Mean and SD indicated. (**o**) Cell death rate by cell state in -EPO culture conditions. Blue points represent bipotent cells. Red points represent E-destined cells (*p < 0.05). Green points represent Mk-destined cells (*p < 0.05, n = 3 movies). Mean and SD indicated. (**p**) Lifespan of cell states by culture condition. Lifespan of cells categorized by state as defined in the time-lapse CFU assays was measured by time between divisions in hours of bipotent MEP (circles), E-destined (squares), Mk-destined (triangles), in control (blue), -TPO (orange), and -EPO (pink) conditions. (*p < 0.05, **p < 0.01, ***p < 0.001, ****p < 0.0001, *ns* not significant, 55 ≤ n ≤ 2035). Mean and SD indicated. (**q**) Total distance of cell states by culture condition. Total distance traveled by cells categorized by state as defined in the timelapse CFU assays of bipotent MEP (circles), E-destined (squares), Mk-destined (triangles), in control (blue), -TPO (orange), and -EPO (pink) conditions. n for each condition and cell state is listed below each graph. *p < 0.05, **p < 0.01, ***p < 0.001, ****p < 0.0001, *ns* not significant, 55 ≤ n ≤ 2035. Mean and SD indicated. (**r**) Diffusion distance of cell states by culture condition. Diffusion distance of cells categorized by state as defined in the timelapse CFU assays of bipotent MEP (circles), E-destined (squares), Mk-destined (triangles), in control (blue), -TPO (orange), and -EPO (pink) conditions. n for each condition and cell state is listed below each graph. *p < 0.05, **p < 0.01, ***p < 0.001, ****p < 0.0001, *ns* not significant, 55 ≤ n ≤ 2035. Mean and SD indicated. (**s**) Peak velocity of cell states by culture condition. Peak velocity of cells categorized by state as defined in the time-lapse CFU assays of bipotent MEP (circles), E-destined (squares), Mk-destined (triangles), in control (blue), -TPO (orange), and -EPO (pink) conditions. n for each condition and cell state is listed below each graph. *p < 0.05, **p < 0.01, ***p < 0.001, ****p < 0.0001, *ns* not significant 55 ≤ n ≤ 2035. Mean and SD indicated.

### Modeling of MEP fate

We performed probability modeling to describe the formation of an Mk/E colony from control MEPs differentiated in MegaCult. As observed through tracking, MEPs may undergo maintenance divisions and expansion divisions interchangeably until exhausting (no longer bipotent) into lineage-destined/committed daughter cells. MEPs may also undergo several rounds of maintenance divisions or expansion divisions until exhaustion. Therefore, the probability of an MEP division outcome does not depend on previous division type until exhaustion, which can be described by a Markov chain (Fig. 4c i). In previous work, Wheat et al. used a homogenous Markov chain model to describe transcriptional state dynamics in hematopoietic stem cells and progenitors^12^. However, tracking indicates that division outcomes change over time, with expansion occurring more frequently during early generations and exhaustion occurring more frequently during later generations (Fig. 2e; Supplemental Fig. 4). Thus, we chose a nonhomogeneous Markov model to account for this time-dependent change in the probability of each division outcome. To create a nonhomogeneous Markov model, we designed a transition matrix with the assumptions that (1) the probability is different for each state transition and (2) the change over time for each transition is also different (Fig. 4c ii). We used the observed sequence of division outcomes from the initial expansion to final exhaustion in each movie, along with the transition matrix, as input to build the nonhomogeneous Markov model. We then applied the model to generate predictions for the probability of a division outcome for an MEP at each generation after plating (Fig. 4d). Comparison between the model and observed data demonstrates a high level of agreement, with the observed MEP data points falling within the 95% confidence interval for each division outcome at each generation (Fig. 4d). The reduced agreement between the model and observed MEP data for the maintenance Mk probability is likely due to the limited number of data points available for establishing the maintenance Mk probability.

### Effects of TPO and EPO on MEPs

To demonstrate the utility of our time-lapse imaging CFU technique, we assessed the roles of EPO and TPO on MEP fate specification. The receptors for TPO and EPO are transcriptionally expressed in the MEP population^1^. To test whether MEP that make Mk/E colonies have different surface expression levels of CD110, the TPO receptor, than MEP that make MK-only and E-only colonies, we used index cell sorting^4^. The data obtained (Fig. 5a) reveal that all MEPs express similar levels of CD110 regardless of the type of colony formed, although there is a trend toward increased CD110 levels in some of the MEP that produce Mk-only colonies (Fig. 5a). Note that analogous studies could not be performed for EPOR with currently available antibodies due to lack of specificity (data not shown).

Using our time-lapse approach on highly enriched human MEP, we evaluated the role of TPO and EPO in MEP lineage specification by culturing MEPs in control conditions replete with the standard cytokine cocktail (recombinant human Interleukin-3, Interleukin-6, Stem Cell Factor, TPO, and EPO), as well as conditions that lacked either TPO or EPO. Based on in situ fluorescent staining with CD41, CD71, and CD235a at day 14 of MEP standard CFU assays (permitting identification of CD71^hi^ E-destined progeny that lacked CD235a expression in the absence of EPO) (Fig. 5b), the lack of TPO or EPO did not affect the relative frequency of bilineage and unilineage colonies (Fig. 5c). However, in the absence of TPO or EPO, there was a decrease in total colony counts (20 ± 2%, p < 0.05) and size (Fig. 5d, Supplemental Fig. 7).

To determine whether the reduced cellularity and total colony number in the absence of TPO or EPO arose from increased cell death, slower proliferation, or less expansion, we utilized the time-lapse CFU approach in conjunction with in situ staining for CD41 and CD71 to measure the cell death, motility, and division rate of bipotent and Mk- and E-destined progenitors grown in control conditions versus conditions lacking TPO or EPO (Fig. 6a–c). We observed that the frequency of E- versus Mk-lineage specification is equivalent for MEPs grown without EPO or TPO (Fig. 6d). However, in the absence of TPO, MEPs underwent fewer expansion divisions compared with control conditions and the absence of EPO (Fig. 6e). MEP self-renewal by maintenance divisions also decreased in the absence of either TPO or EPO compared with control conditions (Fig. 6f). Similarly, MEP exhaustion (loss of bipotency) occurred significantly sooner in the absence of either TPO or EPO compared with controls (Fig. 6g). We confirmed this finding by comparing MEP transitions in the absence of EPO or TPO with the mathematical model (Fig. 6h–k). Taken together, TPO and EPO do not instruct MEP lineage commitment, but do support MEP self-renewal.

MEP cell death also increased significantly in cultures lacking TPO or EPO compared with controls (Fig. 6l). Furthermore, both E-destined and Mk-destined progeny exhibited increased cell death in the absence of TPO and EPO compared with control conditions (Fig. 6m–o). We also observed a significant increase in the time between divisions in cultures lacking TPO compared with control cultures (Fig. 6p). Ultimately, the exclusion of TPO or EPO reduced the proliferative rate and viability of MEPs and thereby diminished the growth of all colony types. The lack of TPO or EPO also significantly stunted the survival of E- and Mk-destined cells, implicating TPO and EPO as important survival factors for MEPs, ErPs, and MkPs.

Unexpectedly, we also observed changes in motility for MEPs and downstream Mk- and E-destined progeny in the absence of TPO or EPO. We measured a significant increase in the total distance traveled by MEPs and E-destined progeny in the absence of TPO and EPO, whereas Mk-destined progeny exhibited an increase in the total distance traveled in the absence of EPO, but a decrease in the total distance traveled in the absence of TPO (Fig. 6q). We found that MEPs cultured in the absence of TPO and EPO also exhibited significant increases in diffusion distance compared with control conditions, whereas E-destined and Mk-destined progeny exhibited an increased diffusion distance only in the absence of EPO (Fig. 6r). Furthermore, MEPs and E-destined progeny exhibited a significant increase in peak velocity in the absence of TPO and EPO, whereas Mk-destined progeny only exhibited an increase in peak velocity in the absence of TPO (Fig. 6s). Taken together, these results suggest that progenitors capable of E and/or Mk differentiation are responsive to TPO and EPO, which regulates the survival, self-renewal and motility of these cells.

## Discussion

Single-cell analyses have enriched our understanding of hematopoietic stem and progenitor cell heterogeneity but have been primarily limited to static or single time-point assays that preclude continuous observation and measurement of changes occurring as cells undergo fate commitment. To measure the dynamics of cell cycling, motility, and cell state, we have developed a detailed approach for time-lapse imaging of CFU assays of isolated hematopoietic progenitor cells over the course of in vitro differentiation at the single-cell level. Using this technique, we have uncovered previously unknown dynamics in bipotent progenitors committing to Mk versus E lineages, including a higher motility of Mk-destined MEPs compared with E-destined MEPs and faster cycling of E-committed cells, which may reflect behavioral phenotypic differences between these progenitors in their native bone marrow microenvironment.

The ability to link cell state (potential) with spatial and temporal measurements of cell cycle, lifespan, and motility enables us to fill knowledge gaps left by static assays, including spatial transcriptomic analyses aimed at identifying cell state by probabilistic inference, and adds to our understanding of the basic regulatory mechanisms underlying lineage commitment.

In the cell culture conditions used, MEP fate commitment was not skewed toward one fate over another. Therefore, we applied a stochastic model to mathematically describe the division outcomes of an MEP as it forms an Mk/E colony. We used an MEP that forms an Mk/E colony as a positive control to ensure that we were observing and modeling a truly bipotent cell. The Markov model is suitable for this application, as its core assumption is that the probability of each event depends only on the previous state, meaning that each transition is “memory-less.” This approach allows us to account for the observation that symmetric expansion divisions can occur after asymmetric maintenance divisions and that the same division outcome can occur multiple times in a row. We can also account for a final state, where the probability of making a state transition after reaching this state is zero. Interestingly, Wheat et al. recently applied the Markov chain model to describe transcriptional state dynamics in hematopoietic stem and progenitor cells^12^. Due to the power of our live cell imaging approach, our tracking results allowed us to extend the Markov model to capture time-dependent changes in probability to model MEP divisional outcomes. This nonhomogeneous approach enables us to accurately model a decreased expansion probability and increased exhaustion probability over time. This change in the probability of self-renewal and maintenance divisions over time is intuitive, as MEPs are not long-term self-renewing stem cells. Interestingly, the consequences of our nonhomogeneous Markov model suggest key facets of MEP biology. In particular, the decision of bipotent MEPs to divide symmetrically or asymmetrically is “memory-less,” as it follows this Markov model. Extending our model with the assumption that MEPs exist as an equal mixture of cells with varying generational age, we would expect approximately 49% of MEPs to exhaust during their first division in culture. This finding offers insight into our historical ratio of MEP colonies, which is approximately 50% Mk/E and 50% E- and Mk-only and suggests that the presence of E-only and Mk-only colonies is due to early exhaustion of MEPs.

We applied single-cell time-lapse analysis to address a long-standing issue in hematopoiesis: the role of the cytokines TPO and EPO in the differentiation of bipotent MEPs. We investigated whether TPO or EPO influence the fate specification, proliferation, survival, or motility of primary human MEPs and their lineage-committing progeny. Index cell sorting revealed similar surface expression levels of CD110 (c-MPL) in MEPs that went on to give rise to E-only, Mk-only, and Mk/E mixed colonies, supporting the hypothesis that TPO is not instructive of MEP lineage commitment. The absence of TPO decreased the number of all three colony types (Mk/E, Mk-only, E-only), without changing their distribution. The absence of TPO also caused a significant decrease in Mk colony size, reflecting the role of TPO in survival and proliferation without influencing commitment to the Mk lineage. Similarly, studies in human pluripotent stem cell-derived bipotent MEPs have shown that TPO drives the expansion and differentiation of Mk-committed cells, as opposed to commitment to the Mk lineage^13^. Studies have also raised the possibility that TPO may bias hematopoietic stem cells toward the Mk lineage via its downstream metabolic effects^13, 14^. Our data do not rule out the possibility that TPO may influence Mk fate commitment of an earlier hematopoietic progenitor cell type. While this is an attractive hypothesis, it remains unclear how TPO would differentially regulate self-renewal versus Mk commitment in hematopoietic stem cells. Furthermore, a lack of TPO accelerates the kinetics of fate specification, resulting from a significant decrease in the frequency of self-renewal divisions (both expansion and maintenance). Although prior studies have proposed that TPO may be critical for the development of MEPs from multipotent progenitors and that FLI1-mediated gene expression downstream of TPO may bias pluripotent stem cell-derived MEPs toward Mk-lineage commitment^15^, these studies started with pluripotent stem cells, not primary bipotent MEPs.

Consistent with prior data suggesting that EPO supports the survival and proliferation of E-committed cells but is not instructive in E-lineage commitment^16^, our results showed that MEPs cultured in the absence of EPO formed colonies containing E-committed cells with a frequency similar to that of controls replete with EPO. However, we observed strikingly smaller E-containing colonies (both E-only and Mk/E) in the absence of EPO compared with the control. Time-lapse CFU analyses revealed that a lack of EPO led to an increased apoptosis rate in lineage-destined progenitors (including Mk-destined progeny), slower division rate, and decreased E-lineage maturation as evidenced by reduced CD235a expression in E-destined progeny compared with control conditions. In cultures lacking EPO, cell death in E-destined progenitors is likely due to EPO dependence, as those E-destined progenitors upregulate EPO Receptor. Importantly, the culture lacking EPO still contained SCF, IL6, IL3, and TPO; thus, this combination of cytokines is sufficient for E commitment of bipotent MEPs.

Overall, we have shown that TPO and EPO play critical roles in the survival and proliferation of Mk- and E-destined progenitors but do not play an instructive role in MEP fate decision in vitro. It has previously been suggested that the action of some cytokines, which relay a generic signal, is lineage-specific because of the unique microenvironment in which the cells arise^16^. It would be interesting to explore whether specific extracellular cues promote the requirement of lineage-specific cytokines, leading to specific differentiation paths. The live-imaging CFU assay described here provides a powerful technique to begin addressing some of these essential issues in hematopoietic biology. Because we assessed only a limited combination of cytokines here, future studies should aim to elucidate how other cytokines may influence fate commitment. Future investigations could focus on bone marrow microenvironment models with our time-lapse cell-tracking method or in vivo conditions to assess whether these additional variables influence progenitor fate decisions.

## Methods

### Flow cytometry and cell sorting

Human granulocyte colony-stimulating factor (G-CSF) mobilized peripheral blood was CD34^+^ enriched (CliniMACS; Miltenyi) and stained with Lineage cocktail-BV510 (BD Biosciences [BD]), CD34-BV421 (Biolegend), CD38^−^ PECF594 (BD), CD45Ra-BV711 (Biolegend), CD135-PE (Biolegend), CD36-PerCPCy5.5 (BD), CD110-APC (BD), and CD41a-APCH7 (BD) antibodies. Human MEPs (Lin^−^CD34^+^CD45Ra^−^CD135^−^CD38midCD110^+^CD36^−^CD41a^−^), MkPs (Lin^−^CD34^+^CD45Ra^−^CD135^−^CD38midCD110^+^CD36^−^CD41a^+^), and ErPs (Lin^−^CD34^+^CD45Ra^−^CD135^−^CD38hiCD110^−^) were sorted on a FACSAria, as previously described^3^.

### Dual Mk/E colony formation assays

MEPs were cultured in MegaCult C Medium Plus Lipids (Stem Cell Technologies) mixed with Collagen solution (Stem Cell Technologies) to a final concentration of 1.2 mg/mL with varying combinations of cytokines as defined in the text including 3.0 U/mL rhEPO, 10 ng/mL rhIL-3, 10 ng/mL rhIL-6, 25 ng/mL rhSCF, and 50 ng/mL rhTPO. All cytokines were purchased from ConnStem (Cheshire, CT) except rhEPO (Amgen).

### Detection of Mk/E colonies with immunocytochemistry

After 12–14 days, fixed and dried cultures were stained using a dual immunocytochemistry assay as previously described^3^. E staining was completed using rabbit anti-GlyA antibody (1:1,500, ABD Serotec, clone YTH89.1), the horse anti-rabbit Impress-horseradish peroxidase secondary kit (Vector Labs) for signal amplification, and nitro blue tetrazolium/5-bromo-4-chloro-3-indolyl-phosphate. Mk staining was completed using mouse anti-human CD41a antibody (BD), goat anti-mouse Impress-AP secondary (Vector Labs), ABC-AP, and Vector Red substrate (Vector Labs). Colonies were scored based on GlyA and CD41a staining as megakaryocyte (Mk) only, erythroid (E) only, or megakaryocyte/erythroid (Mk/E).

### Detection of Mk/E colonies with immunofluorescence

Colonies were stained in situ 10–14 days post-plating with an antibody staining cocktail comprised of Iscove's Modified Dulbecco's Media, anti-CD41-PE or CD41-AlexaFluor 488 (Biolegend), anti-CD235a conjugated to allophycocyanin (APC) (BD), and CD71 conjugated to APC (Biolegend). Colony types were assessed by fluorescence microscopy on a Leica DMI6000 B inverted fluorescent microscope with phase contrast, differential interference contrast (DIC), and epi-fluorescence. Images were acquired with Leica AF6000 digital imaging software and a Cool Snap HQ2 Digital Camera.

**Table:** 

Antibody	Vendor	Catalog #	Dilution
Anti-human CD41-PE	Biolegend	303706	1:500
Anti-human CD41-AF488	Biolegend	303724	1:500
Anti-human CD235a-APC	BD	551336	1:200
Anti-human CD71-APC	Biolegend	334108	1:300
Anti-human CD44-PE	Biolegend	103024	1:300
Anti-human CD36-V450	BD	561535	1:250

### Single-cell indexed colony assays

Individual MEPs were sorted into 96-well plates containing MegaCult C Medium with the cytokines listed above. The flow cytometric parameters used to purify MEPs were recorded on a BD FACSAria and correlated with the colony type formed. Colonies were stained with immunofluorescence antibodies and scored as described above.

### Plating CFU assays for live imaging

Sorted MEPs were thawed and counted using a hemocytometer. For each sample, 50 sorted MEPs were resuspended in 30 µL of MegaCultC medium (Stem Cell Technologies) supplemented with cytokines and mixed with collagen as described above and detailed in the text. A total volume of 15 µL was plated in the center of a MatTek dish (containing a coverslip in place of a plastic bottom for better imaging resolution) and sandwiched under a sterile 12-mm round coverslip to create minimal focal range in the z-axis. Samples were incubated for 45 min to allow for collagen polymerization. An additional 1.5 mL of MegaCultC medium, cytokines, and collagen were added on top of the second coverslip to prevent evaporation and support a long-term culture. Samples were incubated without light exposure at 5% CO_2_ and 37 °C for 24 h before beginning imaging.

### Live imaging

Time-lapse imaging was accomplished with an Olympus (Center Valley, PA) VivaView live imaging microscope. CO_2_ levels were set to 5% and the temperature was set to 37 °C. Images were acquired with a 20 ×/0.75 DIC Olympus U PlanS APO objective with a WD of 0.65 mm. A 0.5 × auxiliary magnification lens was used to produce 10 × magnification per field of view. Excitation light was provided through a liquid light guide from an Excelitas X-cite 110 LED light source, and emitted fluorescence was collected through the following filters: DAPI 435–485; GFP 495–540; YFP 515–535; Cy5 570–625. Image acquisition and processing was done on a high-performance workstation (Windows 7 Professional, 64-bit, Intel Xeon ES-1,620 V3 @3.5 GHz processor, 32 GB memory) with additional 8 TB data drives (Seagate) installed. Images were collected with a Hamamatsu ORCA-Flash4.0 V3 Digital CMOS camera. Using MetaMorph-based VivaView premier acquisition software, images were taken automatically at set intervals. For high exposure cells, cells were imaged every 10 min in DIC starting 24 h post-plating. For low exposure cells, cells were imaged every 2 h from 24–60 h post-plating in DIC followed by every 10 min in DIC (and FITC, TRITC, and Cy5 filtered fluorescent light in designated experiments) for the duration of the experiment, usually 7 days post-plating. Growing colonies were stained in situ with fluorescent antibodies as described above.

### Cell tracking

Images for a given stage position were exported from the VivaView software and adjusted for brightness/contrast, false colored, merged, and concatenated in FIJI. Each stack of images was uploaded to MatLab, stabilized, and automatically tracked using the Baxter algorithm developed by the Blau Lab at Stanford University^11^. The Baxter algorithm was chosen among several cell tracking software packages based on direct comparisons of automated cell segmentation and tracking and was further adjusted to meet the needs of our project. The automated cell tracking was supervised and manually corrected. Quantifiable features of the cells (including cell cycle speed, area, speed of movement, and intensity of fluorescence) in lineage-corrected time-lapse images were measured and exported directly from the algorithm.

### Clustering

Individual cell peak velocity, total distance traveled (µm), time between divisions, and directionality were compared in two dimensions using principal component analysis (PCA). Principal components for each cell were graphed using ggbiplot to add vector labels for each of the cell parameters. Ellipses were added representing the 68% confidence interval, or one standard deviation, for each cell type compared by PCA. Clustering of the principal components for each cell was accomplished first through hierarchical clustering, which automatically determined the appropriate number of clusters to use for *k*-means clustering^17^. *k* = 3 clusters was used to define *k* for *k-*means clustering and subsequent coloring of cells in the PCA plot. PCA analysis, clustering, and graphs were generated using R.

### Modeling

Sequences of cell divisions were created by linking cells from the last frame of the parent and the first frame of the subsequent daughter. Sequences were created between the starting cell in each control MEP movie and the final exhaustion of each daughter cell. These control sequences were then used to build a nonhomogeneous Markov model using the ‘nhm’ package and functions in R^18^. Visualization of the possible transitions between division types was accomplished in MATLAB using the ‘dtmc’ function from the ‘Econometrics Toolbox.’ To build the model, a matrix was generated that allowed for transitions between expansion and maintenance divisions to occur with unique probabilities. The matrix was also designed to create an absorbing state in the exhaustion division, as this division represents differentiation and loss of MEP lineage potential. The same logic was used for the design of a matrix representing the change over time between each transition. A Weibull distribution was selected as the basis for the type of model to fit. The Weibull distribution is typically used to model life data and time to failure, which can be thought of as time to exhaustion in our application^19^. Predicted probabilities and 95% confidence intervals for the type of division at each generation were determined using the ‘predict.nhm’ function and were calculated for 13 generations, as that was the maximum number of generations tracked in the dataset.

### Data storage/management

Average file sizes for each stage position reached approximately 1 GB. Files and backups were stored on 8 TB spinning disk hard drives (Seagate) and a cloud-based backup system.

### Human subjects

All work was conducted according to the Declaration of Helsinki principles. Collection and use of human cells were approved by the Yale University Institutional Review Board. Healthy donors who were already donating cells for allogeneic transplantation provided written informed consent prior to use of surplus G-CSF mobilized cells for research.

### Statistical analysis

Data are plotted as mean plus standard deviation. Statistical analyses were performed using an analysis of variance or Student’s *t* test with a p-value ≤ 0.05 considered statistically significant.

## Supplementary Information

Supplementary Legends.

Supplementary Information 1.

Supplementary Information 2.

Supplementary Video 1.

## Data Availability

All data generated or analyzed during this study are included in this published article and the supplementary information files.
