# Supplementary material for: Multiparameter analysis of timelapse imaging reveals kinetics of megakaryocytic erythroid progenitor clonal expansion and differentiation
Source: Sci Rep. 2022 Sep 28;12:16218. doi: 10.1038/s41598-022-19013-x (PMC9519589; doi:10.1038/s41598-022-19013-x)
Supplement: Supplementary file 1 — Supplementary Legends. [file 41598_2022_19013_MOESM1_ESM.docx]

**Supplementary Figure Legends**

**Supplemental Table 1: Antigen temporal expression patterns in Mk/E progenitors.** Temporal detection by microscopy of surface expression by antibody-mediated immunofluorescence is reported for all tested antigens at day 1, 7, and 14 post-plating.

**Supplemental Fig. 1: Optimization of live imaging.**

**a,** Time to first division post-plating of Mk/E progenitors**.** MEPs grown in control conditions, or conditions lacking TPO or EPO, as well as sorted ErPs and MkPs were plated in timelapse CFU assays. The time to first division is reflected in the bar chart. n ≥ 4. Mean and SD indicated.

**b,** Antigen temporal expression patterns in MEP CFU assays**.** Representative clonally growing cells at day 0, 7, and 14 post-plating were stained *in situ* and temporal expression patterns were captured. Scale bars are set to 50 µm for day 0 and 250 µm for days 7 and 14.

**Supplemental Fig. 2: Baxter algorithm cell segmentation settings.** Cell segmentations are imported from the nested U-Net segmentation code we developed into the Baxter algorithm for downstream lineage tracking analysis.

**Supplemental Fig. 3: Baxter algorithm cell tracking settings.** Tracking settings for automated tracking of single cells in timelapse CFU assays. Code available @ https://github.com/klasma/BaxterAlgorithms

**Supplemental Video 1: Representative timelapse sequence of MEPs growing for 7 days in CFU conditions with *in situ* staining.** Images were acquired every two hours for the first 36 hours, and every ten minutes thereafter. Green fluorescence indicates cells expressing CD41 (megakaryocyte marker) that were stained with a fluorescently conjugated antibody *in situ*. Red fluorescence indicates cells expressing CD71 or CD235a (erythroid markers) that were stained with a fluorescently conjugated antibody *in situ*. The cells are marked with false colored dots corresponding to the lineage tree in which blue cells are upstream of both megakaryocytic and erythroid progeny demonstrating their functional bipotency, red cells are upstream of only erythroid progeny, and green cells are upstream of only megakaryocytic progeny.

**Supplemental Fig. 4: MEP division rates and outcomes.**

**a,** Frequency of division outcomes by generation in culture**.** Frequency of MEP divisions normalized to total number of MEP divisions at each generation in culture was categorized as expansion (blue: 1 MEP -> 2 MEP), maintenance (purple: 1 MEP -> 1 MEP + 1 E- or Mk-destined progenitor), or exhaustion (yellow: 1 MEP -> 1 E-destined progenitor + 1 Mk-destined progenitor) normalized to total MEP divisions (n = 6 movies). Mean and SD indicated.

**Supplemental Fig. 5: Motility is a differential behavioral phenotype between progenitor cell transitioning states.**

**a,** Peak velocity by cell state and generation in culture**.** Single peak velocity of cell states of bipotent MEP (blue; n = 188), E-destined (purple; n = 964), Mk-destined (orange; n = 49), committed ErP (red; n = 505), and committed MkP (green; n = 47) cells and grouped by generation in culture (for MEPs, ErPs, and MkPs) or generation since blue (for lineage-destined cells). Mean and SD indicated.

**b,** Directionality by cell state and generation in culture. Directionality of cell states of bipotent MEP (blue; n = 188), E-destined (purple; n = 964), Mk-destined (orange; n = 49), committed ErP (red; n = 505), and committed MkP (green; n = 47) cells and grouped by generation in culture (for MEPs, ErPs, and MkPs) or generation since blue (for lineage-destined cells). Mean and SD indicated.

**Supplemental Fig. 6: Validation of k-means clustering.** K-means clustering was validated by generating hierarchical clustering of cells tracked in the timelapse CFU assays based on quantified behavioral phenotypes: lifespan, total distance traveled, directionality, and peak velocity.

**Supplemental Fig. 7: Mk-only colony area in control, -TPO, and -EPO CFU conditions.** Mk-only colony area was measured in control, -TPO, or -EPO CFU assays from images analyzed in FIJI. n ≥ 3, *p < 0.05. Mean and SD indicated.

**Supplementary Dataset: Multiparameter single cell dataset.** Each row represents a single cell measured from timelapse images. Columns represent the exported measurements made in the Baxter Algorithm (inferred cell state, ancestry, frames and positions of cells) and calculated values representing lifespan, motility, and division outcomes.
